# Description of Various Factors Contributing to Traffic Accidents in Youth and Measures Proposed to Alleviate Recurrence

**DOI:** 10.3389/fpsyt.2017.00094

**Published:** 2017-06-01

**Authors:** Ludovic Gicquel, Pauline Ordonneau, Emilie Blot, Charlotte Toillon, Pierre Ingrand, Lucia Romo

**Affiliations:** ^1^University Center of Child and Adolescent Psychiatry, Clinical Research Unit, Henri Laborit Hospital Center, Poitiers University, Poitiers, France; ^2^Faculty of Medicine and Pharmacy, Biostatistics Department, Poitiers University, Poitiers, France; ^3^EA 4430 Paris Ouest Nanterre la Défense University, Nanterre, France; ^4^Sainte Anne Hospital Center, INSERM Unit U-894, Paris, France

**Keywords:** traffic accidents, recurrence, young drivers, adolescence, prevention, therapeutic programs

## Abstract

Traffic accidents are the leading cause of hospitalization in adolescence, with the 18–24-year-old age group accounting for 23% of deaths by traffic accidents. Recurrence rate is also high. One in four teenagers will have a relapse within the year following the first accident. Cognitive impairments known in adolescence could cause risky behaviors, defined as repetitive engagement in dangerous situations such as road accidents. Two categories of factors seem to be associated with traffic accidents: (1) factors specific to the traffic environment and (2) “human” factors, which seem to be the most influential. Moreover, the establishment of a stronger relation to high speed driving increases traffic accident risks and can also be intensified by sensation seeking. Other factors such as substance use (alcohol, drugs, and “binge drinking”) are also identified as risk factors. Furthermore, cell phone use while driving and attention deficit disorder with or without hyperactivity also seem to be important risk factors for car accidents. The family environment strongly influences a young person’s driving behavior. Some interventional driving strategies and preventive measures have reduced the risk of traffic accidents among young people, such as the graduated driver licensing program and advertising campaigns. So far, few therapeutic approaches have been implemented. Reason why, we decided to set up an innovative strategy consisting of a therapeutic postaccident group intervention, entitled the ECARR2 protocol, to prevent recurrence among adolescents and young adults identified at risk, taking into account the multiple risk factors.

## Introduction

Traffic accidents are the leading cause of death among young people aged 15–29 years in industrialized countries (1). For example, in Britain in 2011, 22% of road accidents involved at least one young driver aged 17–24 years. In fact, accidents including young drivers typically represented about a quarter of all deaths on the road (2).

In France, traffic accidents also represent a major and persistent public health problem. They are by far the leading cause of death among young people aged 15–24 years (3). A global process of reducing fatal traffic accidents has been applied for all age groups since 1979. According to an annual report from the Organization for Economic Co-operation and Development (4), a 15% decrease in the number of road deaths was observed between 2010 and 2014, a drop similar to the one observed period between 2006 and 2010.

However, this declining trend is still fluctuating. In fact, in January 2016, the French Ministry of the Interior stated that road deaths were up by 2.4% in 2015, for the second consecutive year, while the number of injury accidents has declined by 3.6%.

**Figure 1:**
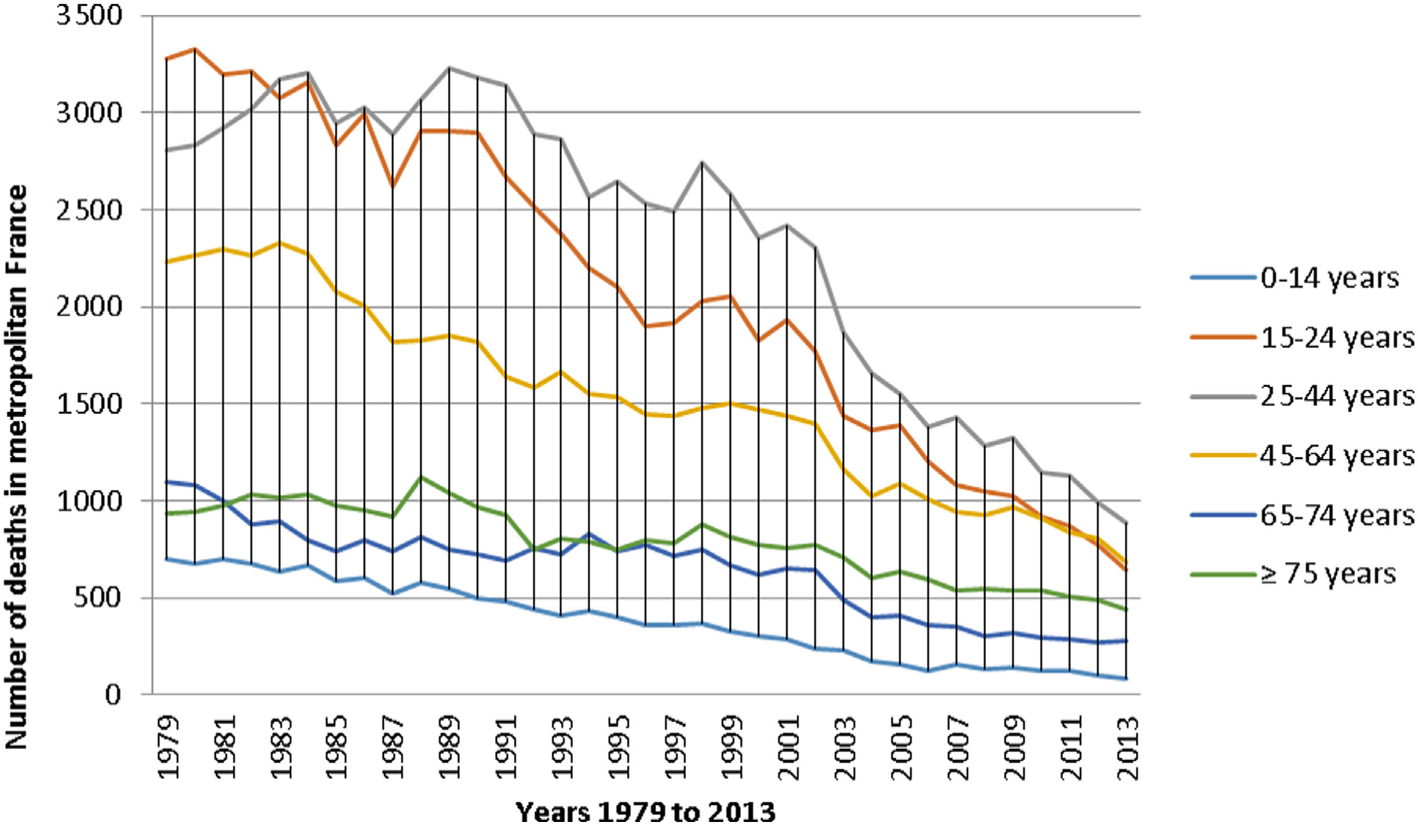
**Evolution of the number of traffic accidents deaths for each age group in France from 1979 to 2013 (5)**.

Since 1979, traffic accidents fatalities mainly affect three age groups (5) (Figure 1).

**Figure 2:**
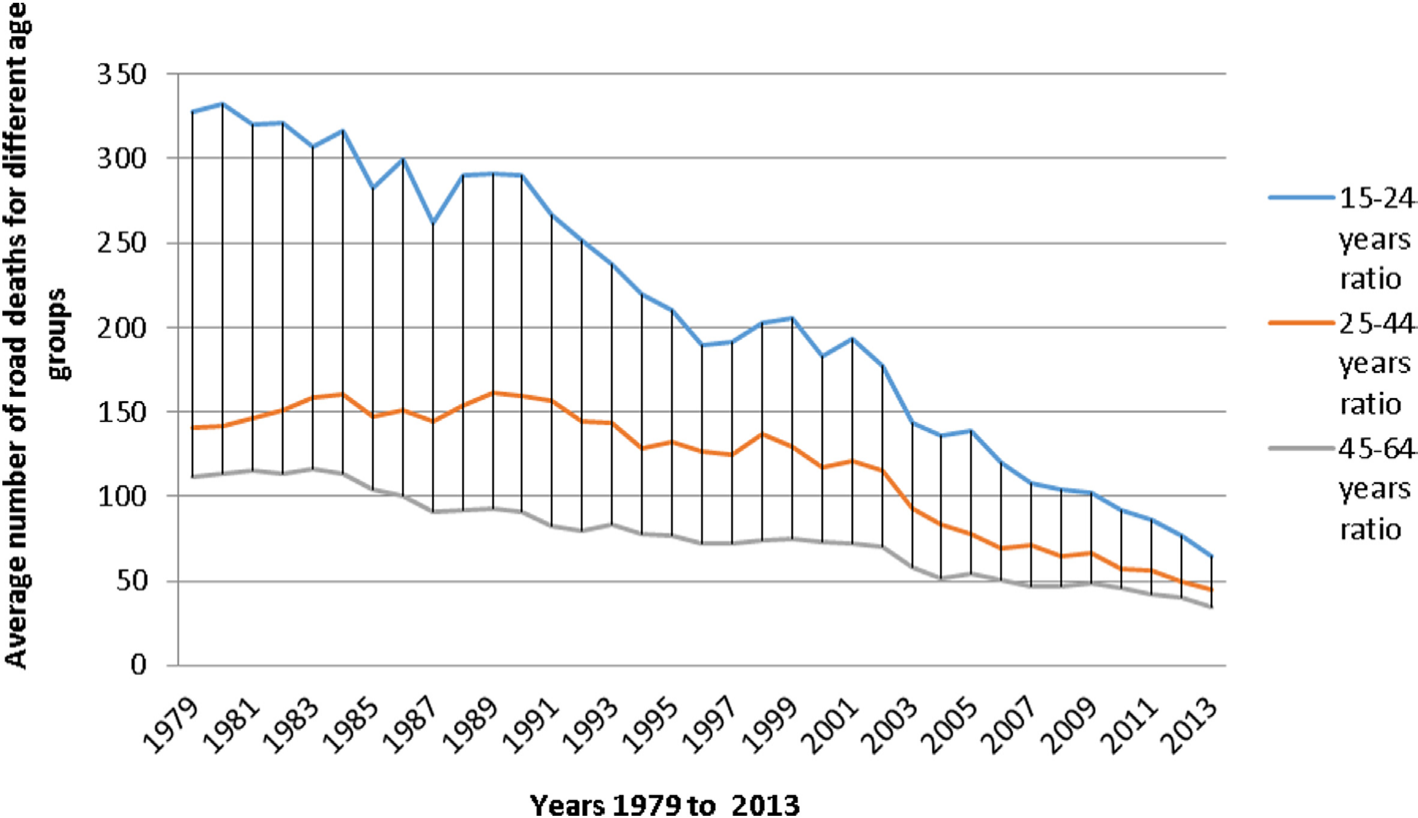
**Average road deaths ratio in France for different age groups between the years 1979 and 2013 (5)**.

Furthermore, according to the French epidemiological center on the medical causes of death (5) (Figure 1), the age group 25–44 years seems to be the most affected by road fatalities. However, when divided by the number of constituent years of each age group, the 15–24 age group had the highest average mortality rate (Figure 2). Indeed, in view of the nine constituent years of the 15–24 age group, the number of accidents is greater than the 19 constituent years of the other two age groups (i.e., 25–44 age group and 46–64 age group).

The 15–24 age group represented alone about 20–30% of deaths from traffic accidents, making it the most affected age group by road fatalities. According to the data from the French ONISR (6), young people aged 18–24 years still contributed to 17% of road deaths in 2014, although only representing 8% of the population. Hence, they seem to be twice as likely as other age groups to lose their lives on the roads.

Moreover, according to the International Road Traffic and Accident Database (7), young French adults aged 18–24 years have among the highest mortality and morbidity rates in European countries.

The high levels of risks faced by young drivers are the product of both their own developmental characteristics and their specific environment.

## Traffic Accidents, Adolescence, and Brain Maturation

According to Courtois (8), risk taking is normal during adolescence, a distinguished period of an individual’s development. It is indeed a phase during which neurological data display a significant maturation of the frontal and temporal lobes. According to Dayan and Guillery-Girard (9), adolescent behaviors (impulsivity, sensation seeking, and risky behaviors) are more frequent between 15 and 25 years, reaching a maximum at 18 years and decreasing after 25 years. These behaviors are related to a major brain reorganization that selectively affects the prefrontal cortex. The specific adolescent behaviors could be the consequence of cognitive control impairment due to this brain maturation. Thus, the cortical structures involved in high-level decision-making processes (prefrontal cortex) become transiently immature. Barbalat et al. (10) imply that adolescents tend to choose riskier options because they feel less risk aversion than adults and devaluate the future consequences of their choices.

From an economic and neurologic point of view, some studies have shown that two cerebral areas are implicated in risk taking (10) as follows.

– The anterior cingulate cortex and the posterior prefrontal cortex whose function is to optimize decision-taking. It allows to normally take advantage of previous unfavorable outcomes and to better manage conflicts between the different choices.
– The insular cortex involved in negative affects such as disgust. It inhibits risky decision-making.

Furthermore, Mantyla et al. (11) conducted an experiment on students to determine whether the development of the executive control system located in the prefrontal lobe could be associated with driving performances. For that purpose, they evaluated young people’s driving performances with a driving simulation test and six other experimental tasks. Results showed that individual differences in brain maturation had an impact on driving performance during the simulation task.

Various studies have shown that the dysfunction of these brain regions in adolescents was significantly correlated with behavioral measures of risk taking during the last MRI task. These risk behaviors are as much about substance use, sports accidents as they are about road accidents. Coslin (12) stresses that all risk behaviors reveal a common characteristic: whatever causes them, these conducts can be very destructive for one’s self as for others and must be taken seriously. According to this author, the sought sensations associated with these behaviors will tend to be self-reinforcing. Repeated sensation seeking is hypothesized to fill the narcissistic void experienced by a young person passing through an identity crisis, particularly, sensations associated with speed and risks taken on the road.

## Traffic Accidents and Their Risk in Adolescence

Adolescence is a transitional period marked by a more in-depth environmental exploration than by safety seeking. Thus, according to Courtois (8), “the most vulnerable adolescents are more likely to implement more dangerous and/or less structured risk behaviors.” In fact, for teens entering the new world of adults, their poor knowledge of rules as well as high-risk behaviors increases their frequency of hazard exposures (13).

According to Michel et al. (14), risk behaviors are established during adolescence and are defined as a deliberate and repetitive engagement in dangerous situations. They are also divided into two types of risk: short-term and long-term risk. The first type leads to behaviors involving the notion of acts and falls directly into the somatomotor register. Here, the alternative is restricted to either victory or failure in terms of accident or death. As for the second type, it reflects the potential danger that occurs in duplicating an activity, such as the one occurring during the repeated use of psychoactive substances. Furthermore, these authors also identified a risk based on the individual’s participation in his activity, particularly when it comes to the driver’s choice to take risks or not. He could decide to violate safety rules, and in this case, risk taking is considered active and does not solely depend on external factors such as other motorists’ driving styles. Thus, risk taking is a decision that involves a choice characterized by a certain degree of uncertainty about the probabilities of failure or success. In fact, Chumpawadee et al. (15) found that Thai students who have engaged in risky behaviors during motorcycle driving were more likely to view these behaviors as normal, in addition to being less likely to have adequate self-control. These authors also found that having a greater awareness of motorcycle accident risk behaviors was significantly associated with a lower risk of engagement in risky behaviors.

Moreover, Pérez-Diaz (16) suggested that it was not only about risk exposure, assessed by the time spent on the road and the type of vehicle, but also about a much more complex genetic predisposition to risk. In fact, it takes into account each person’s individual aspects (sensorimotor and intellectual abilities, hyperactivity, aggressiveness, impulsivity, psychopathological aspects such as antisocial behavior trends, etc.), in addition to environmental aspects (influence of family background, peers, etc.). Additionally, Higelé and Hernja (17) concluded that the emotional and psychological components of young drivers’ behavior, particularly through their relation to risks, were nowadays identified as being the main causes of these drivers’ big involvement in traffic accidents.

## Traffic Accidents, Adolescence, and Risk Factors

### Accidents and Individual Risk Factors

#### Accidents and Personality Dimensions

There seems to be two categories of factors associated with car accidents (18). On one hand, there are factors specific to the traffic environment and the vehicle in question, and on the other hand, there are factors linked to “human” determinants (i.e., the driver himself). Generally, the majority of car accidents seem to be associated with the “human” factor. Irritability and anger, for example, are factors included in the latter category. Many researchers have implied that these factors greatly undermine road safety and highlighted their important contribution to car accidents. Along these lines, Chliaoutakis et al. (18) suggested that high involvement of young drivers in car crashes was associated with having no tolerance on the road, getting easily (or without serious reason) irritated, expressing aggression or hostility toward other drivers, not being able to cope with stress, and not being able to control his/her emotions. These results are complementary to those of Norris et al. (19) who found that future motor vehicles accidents were strongly predicted by high hostility combined with poor self-esteem.

Javadi et al. (20) aimed to determine how each of the personality factors, including mental health, depressive disorders, self-esteem, aggression, and parenting, contribute to traffic accidents of young boys aged between 18 and 24 years old. The authors explain that among the mental health aspects, depression is a type of negative emotion, which might negatively affect the driver’s interpretation of the traffic environment, his/her driving behaviors and concentration, so that he/she cannot react and behave properly in the required circumstances. Depressed mood and failure schema (which means that failure is inevitable for the individual) could predict violations and mistakes in boys. The authors suggest having a better understanding of the contributing factors in adolescent’s driving behaviors and early interventions toward high-risk driving behaviors can help prevent life-threatening consequences in adulthood.

Furthermore, the study by Vassallo et al. (21) showed that the strongest correlations associated with risky driving patterns were antisocial behavior, excessive alcohol consumption, and relational status. Gender, school completion, temperament, civic engagement, and antisocial peer relationships were also correlated with the different patterns of risk behavior.

Moreover, Waylen and McKenna (22) found that risky attitudes toward road use were observed in adolescents long before they learned to drive. Thus, this shows that a driver’s risky behaviors are not only the function of being in control behind the wheel but are also determined by individual characteristics. Hence, in order to be effective, preventive interventions must target young people before they are old enough to be allowed to drive.

In their review, McDonald et al. (23) identified poor hazard anticipation skills as factors determining young drivers’ car crash risk. Few training programs have been developed to improve these skills. All studies included in this meta-analysis found that young drivers showed an improvement in anticipating risk. However, none of these studies assessed their long-term effects on road accidents.

#### Accidents and Relation to Speed

Michael et al. (24) evaluated young two-wheeler riders’ speeding inclination and other factors linked to it including general attitude to riding, riding behaviors (i.e., engaging in competition and stunts), emotional states associated with riding, motives associated with riding fast, sensation seeking, traffic violations, age, gender, years of riding, riding frequency, and self-report of riding speed. They found that men reported significantly greater inclination to speed than women and that more years of riding were linked to a greater tendency for riding fast. These results are in accordance with the results by Styles et al. (25) who found that more driving years increased drivers’ confidence and thus increased their risk taking while driving. Rathinama et al. (26) found similar results in young motorcyclists (aged between 10 and 16 years): the more the child motorcycle rider had experience, the higher was his traffic accident risk. They also found that 35% of boys did not respect safety distances from other vehicles and that 20% of them were already involved in an accident. Michael et al. (24) also pointed out that speeding was related to the emotional states it triggered. In short, driving fast seems to be influenced by psychological factors such as beliefs and perceptions associated with speeding, motives for speeding, and perceived speed and safety. Nevertheless, it is also influenced by behavioral factors such as sensation seeking, risk taking, the desire to reduce travel time, stress, and affect states.

#### Accidents and Consumer Behaviors

##### Alcohol Consumption

Previous studies have widely examined the effects of alcohol and drugs on traffic crashes. They highlighted the strongly negative impact the abuse of these substances had on road accidents’ incidence.

The SAM study [short for “drugs and fatal accidents” by Elsande et al. (27)] found that the risk of being held accountable for a fatal accident appears to be multiplied by 8.5 times among drivers with a blood alcohol level over the legal limit. This added risk is all the more important given that alcohol consumption is associated with cannabis consumption; here, the risk appears to be multiplied by approximately 15 times. Interestingly, 24% of fatal accidents in 2014 involving individuals aged 18–29 years are associated with the driver having consumed alcohol (28). In this manner, as regularly specified by the French ONISR in its annual reports, alcohol consumption contributes to high rates of fatal accidents mainly caused by speeding, drug consumption, and non-use of seat belts.

According to Waylen and McKenna (22), alcohol greatly increased the probability of having a car accident and the severity of its consequences. Espada et al. (29) added that the occupant of a vehicle is three times more likely to die of a fatal injury after a car accident if having consumed alcohol compared to being sober. In fact, alcohol could impair driving capacities and cause accidents and/or collisions. Indeed, Dang et al. (30) demonstrated a relationship among the precociousness of the first alcohol intoxication and risky behaviors on the road among young drivers. Al-Abdallat et al. (31) showed that alcohol use significantly reduced a person’s motor skills due to its impact on concentration, alertness level, and reflexes. Brubacher et al. (32–34) found that the risk of car crashes increased with alcohol use and was higher among young drivers. Thus, alcohol seems to be the leading cause of road accidents, being involved in one-third of serious crashes. These authors also found alcohol in 17.8% of injured British Columbia drivers and cannabis in 12.6% of them. Both these substances have been associated with a decrease in psychomotor skills required for safe driving.

It is important to mention the emergence of a particularly singular alcohol consumption among young people: the “binge drinking” phenomenon. In fact, recent European surveys showed an increase in alcohol and cannabis use among teenagers (33, 34). According to the World Health Organization (35), the harmful use of alcohol accounts for a substantial portion of the global burden of disease, in addition to being the third highest premature death risk and disability factor worldwide. In 2004, more than 2.5 million people worldwide have died from alcohol-related causes, including 320,000 young people aged 15–29 years. These studies pinpointed the common increase among young people of a pattern of excessive alcohol drinking associated with euphoric effects (36). This phenomenon is better known as “binge drinking,” a model defined by consuming a maximum amount of alcohol in a minimum time period. It is a different from any other practice related to alcohol consumption. Buelga and Musitu (36) found that 36% of Portuguese and 89% of Danish teens aged 15–16 years had already experienced drunkenness. The effects of this excessive-episodic drinking can lead to very serious brain damage. In their study on 121 participants aged 18–25 years, Bo et al. (37) assessed the effects of binge drinking on cognitive performance. They used the three last questions of the Alcohol Use Questionnaire and combined them into a binge score that was entered as a predictor of cognitive performance. They found that binge drinking significantly predicted faster reaction times and impairment in response adjustment.

##### Consumption of Psychoactive Substances

Alcohol and drug use are known to increase the risk of traffic crashes, especially among youth (38). Over the past two decades, the prevalence of cannabis and alcohol use in drivers involved in fatal car crashes has increased approximately fivefold from below 2% in 1991 to above 10% in 2008 (39).

In France, despite taking early security measures to decrease drunk driving, the first decree for the systematic search of narcotics among drivers involved in serious road crashes did not appear before 2001. This gap between reforms against alcohol and those against drugs can be explained in several ways. On one hand, society’s awareness of the dangers of certain drugs (such as cannabis) took longer to acknowledge than those of alcohol. Indeed, the impact of alcohol’s effects could be analyzed as it was listed in the bulletins of accidents indexed by the police for every accident (fichiers BAAC, *Bulletin d’Analyse des Accidents Corporels de la Circulation*, 2005). This was not the case with drugs where information was not available immediately. On the other hand, the resources needed to detect these substances have been placed into service well after those used to test blood alcohol level.

In the study conducted in Jordan, Al-Abdallat et al. (31) showed a link between the use of alcohol and psychotropic drugs and increased road accidents’ risks. This is due to the effects of these drugs on the central nervous system by impairing a driver’s intellectual functions, judgment, and reflexes. Similarly, the results of the study by Dang et al. (30) showed that factors associated with risky driving behavior are, in boys, having a degree, sports practices, involvement in a fight in the last year, as well as the precocity of cannabis consumption. The latter being one of the major factors involving driving risk taking in girls, with entry into sexuality.

According to a meta-analysis conducted by Gjerde et al. (40), it seems that the combined use of two or more psychoactive substances was significantly associated with higher risks of traffic crashes. In fact, the biggest increase of road accidents was observed when alcohol and drugs were simultaneously consumed. Furthermore, Dubois et al. (39) found that drivers who were positive for cannabis alone had a 16% increase in the odds of an unsafe driving action. However, these odds increased by approximately 8–10% when alcohol and cannabis were combined.

The association between the simultaneous consumption of drugs and higher traffic accidents could be explained by the fact drug use induced greater involvement of youth in other risky behaviors (i.e., night driving, driving in the snow, constant change of radio station, driving above the speed limit) (38).

#### Accidents and Attention Disorders

##### Attention Deficit Disorder with or without Hyperactivity

Alcohol and drugs are main factors contributing to accidents, such as highlighted above. However, in Europe and the United States, the greatest cause of deaths seems to be due to unintentional injuries. The risk of these injuries is increased with the presence of an attention deficit hyperactivity disorder (ADHD) (41). Overall, the risk of death is doubled compared to individuals without ADHD, and this risk is even higher 1 year after diagnosis. In fact, the occurrence of this diagnosis in adulthood significantly increased the risk of death compared to its diagnosis in childhood or adolescence. According to Dalsgaard et al. (41), ADHD is closely linked to the use of psychoactive substances. Thus, the combination of ADHD and substance abuse can be particularly dangerous and could potentially increase the risk of a traffic accident and its recurrence. Indeed, Vingilis et al. (42) found that the majority of patients with an ADHD had associated problems such as psychological distress, antisocial behavior, anti-anxiety and antidepressant medication use, substance use disorders, and social problems. Thus, these authors have shown that the status of antisocial personality disorder and cannabis use is significant predictors of road accidents or at least negative driving-related outcomes when associated with ADHD.

Additionally, it seems that teenagers with ADHD are more prone to all sorts of accidents compared to the ones without ADHD (43). Clancy et al. (44) tried to determine whether ADHD adolescents, aged 13–24 years, showed more unsafe road-crossing behaviors than healthy controls. They found that individuals with ADHD had a lower margin of safety and evidenced twice as many collisions as compared to controls. In their study, El Farouki et al. (45) hypothesized that ADHD increased the effect of external distractions and traffic crash responsibility. They found that ADHD and external and/or internal distractions were important factors leading to a higher risk of traffic crashes and injuries. This could be due to the fact that participants with ADHD had a greater difficulty in managing the dual task situation, in addition to having insufficient attention and inadequate behavioral responses. It can also be explained by the impulsivity of adolescents with ADHD and their motor coordination difficulties (43). Furthermore, in their meta-analysis of behavioral outcomes and a review of effect size of pharmacological studies, Jerome et al. (46) found that patients with ADHD committed significantly more traffic violations, had less safe driving habits, and were more involved in traffic accidents compared to controls. It could be due to the fact that adults and adolescents with ADHD seem to be more prone to driving anger (aggressive driving), aggression, impulsivity, risk taking, and driving under the influence of alcohol and/or drugs. Nevertheless, some of these results should be interpreted with caution. In fact, another recent meta-analysis by Vaa (47), based on 16 accident studies, showed that all these studies failed to confirm that ADHD drivers had more drunk driving than drivers without ADHD. Smorti and Guarnieri (48) evaluated the contribution of impulsiveness and aggressive and negative emotional driving to predict traffic violations and accidents, taking into account potential mediation effects. They concluded that impulsiveness was not associated, neither directly or indirectly, with traffic accidents. However, it modulated the behavioral and emotional states of young drivers while driving, which in turn could have influenced risky driving.

Consequently, according to Sargant and Finlay (49), health professionals should encourage the management of anger, frustration, and irritability, in addition to implementing an adapted and systematic treatment to decrease the risk of accidents among young people with ADHD.

##### Mobile Phone Use

The use of a mobile phone has previously often been associated with positive outcomes such as allowing long distance communication (50). However, with time, its use has increasingly been associated with harmful or problematic behaviors. Indeed, according to the French ONISR (51), this emerging factor could be responsible of 25–50% of injury accidents. Additionally, the collective expertise of the French institute of science and technology for transport, development, and networks (52) and the French institute of health and medical research (53) claimed that a phone call could triple the risk of road accidents. In France, the first decree to forbid the use of handheld phones while driving, considered to distract the driver, came into effect in 2003. Many studies have assessed the negative impact of mobile phone use on driving skills. Strayer et al. (54) showed that driving impairments associate with using a mobile phone could be as profound as those associated with driving while drunk.

Saifuzzaman et al. (55) found that mobile phone use while driving was a significant distraction, especially in young drivers, that impaired driving performance, thus becoming a leading cause of traffic motor vehicle crashes. For example, drivers were more likely to miss traffic signals (stop signs, traffic lights, etc.) and were involved twice as often in car crashes when having a phone conversation while driving. Generally, drivers maintained slower driving speed, larger vehicle spacings, and had longer time headways when engaged in phone conversations. This could suggest possible risk compensatory behaviors associated with phone conversations while driving, or it could be the consequence of the distraction itself on driving performance. Finally, the general conclusion on the effects of mobile phone use while driving suggests that both the use of handheld and hands-free mobile sets significantly increased the risk of having a car accident (56). This is in accordance with the results of the meta-analysis conducted by Caird et al. (57), concerning the effects of cell phones on driving performance. They found that the use of either phone types mentioned above was associated with a mean increase of 40% of reaction time and an accident risk multiplied by 4.

According to Billieux et al. (58), it seems that mobile phone use while driving is associated with a high level of sensation seeking *via* impulsive and dangerous behaviors. Thus, in situations in which the driver needs to concentrate, the consciousness of the risks arising from the situation (i.e., phoning while driving), is likely to create intense excitement.

### Accidents and Family Risk Factors

#### Accidents and Family Climate

Additional research tried to identify behavioral and non-behavioral factors among adolescents in relation to traffic accidents. In their longitudinal study on patterns of adolescent psychosocial behavior and substance use of risky driving groups, Bingham and Shope (59) used the Problem Behavior Theory to model risky driving among adolescents and young adults’ risky driving from adolescent problem behavior. They found that characteristics of young adults in the riskiest driving groups included a low level of parental monitoring, an increased parental permissiveness, and a weaker social bond. In fact, these developmental traits seem to identify individuals who are likely to endanger themselves and others through risky driving. They are the ones who should receive early interventions to reduce the likelihood of subsequent risky driving.

Furthermore, Taubman-Ben-Ari and Katz-Ben-Ami (60) evaluated family climate in relation to road accidents in adolescents in four different studies. In their first study, they used the “family climate for road safety scale” (FCRSS) to assess seven aspects of the parent–child relationship (i.e., modeling, feedback, communication, monitoring, non-commitment, messages, and limits) and reported the associations between these factors and dangerous driving. In their third study, they found that positive correlations between the FCRSS and youngsters’ reported proneness to take risks while driving. These factors were in fact positively associated with various dimensions of family functioning. Finally, in their second and fourth study, they found significant associations between the FCRSS factors and both driving style (risky, angry, anxious, careful) and family cohesion. These studies eventually showed that young drivers who perceived their parents as good role models, encouraging autonomy and commitment to safety, and setting clear limits on the infringements of the highway code, tended to take less risks while driving and drove more carefully and less aggressively. However, teenagers who did not perceive their parents as guarantors of their safety may take more risks (61). These studies highlighted the importance of both family climate and environment in a person’s risk taking.

These results are in accordance with the study by Sabaté-Tomas et al. (62), who found that family and peers were the most influential factors on the creation of a high-risk profile in young drivers. In addition, driving schools seemed to be the strongest protective factor in preventing the appearance of risky driving profiles. The authors explained that the same driving behavioral pattern was generally repeated in at least two generations in the same family in both the low- and high-risk young drivers’ groups. Consequently, high-risk drivers maintained the reckless driving behavior their parents had, whereas low-risk drivers had attitudes to road safety similar their parents.

Curry et al. (63) found that interventions targeting parents and directed toward parents’ cognitions, behaviors, and skills allowed the improvement of parental supervisory behaviors during the learning driving stage and at the start of the independent driving stage. These interventions also promoted teen driver’s skills acquisition and reduction of their risky driving behaviors.

## Preventive Therapeutic Strategies for Recurrence of Traffic Accidents in Adolescence

### Current Preventive Strategies

#### Example of Political Measures: The Graduated Driver Licensing

Accidents involving young drivers resulted in significant morbidity and mortality in France and other countries such as Great Britain, the United States, etc. (64). In the last few years, the GDL program was developed and used in some countries, like United States, Great Britain, Canada, South Africa, and Australia, in order to address this scourge. It allowed drivers to gain experience in low-risk driving conditions by adding an “intermediate” phase between the learning stage and the acquisition of the driving license. This new licensing program required young drivers to advance through several stages where they were subject to a variety of restrictions that reduced their exposure to high-risk driving conditions (i.e., adult supervision, daytime driving, passenger limits, etc.) (65). Kaafarani et al. (66) aimed to determine the effect of this 2007 law on the incidence of total motor vehicle crashes and accidents they called “fatal” motor vehicle crashes among drivers aged between 16 and 29 years, divided into three age groups (16–17, 18–20, and 25–29 years). They found that total motor vehicle crashes significantly decreased following the law for all three age groups, with a greater decrease in the 16–17 years (37%) and the 18–20 years (25%) compared to the 25–29 years (15%). The rates of fatal motor vehicle crashes also significantly decreased in all three groups. Thus, it seemed that this type of political measure was able to decrease the accident rates among youth.

Moreover, the effectiveness of the GDL programs varied according to its components. Chen et al. (67) showed that the comprehensive GDL programs were associated with a 20% reduction in 16-year-old drivers’ fatal crash involvement rates. The greatest benefit appeared to be associated with programs that included age requirements and a waiting period of 3 months and above before the intermediate stage, nighttime driving restriction, and either 30 h or more of supervised driving or passenger restrictions.

In an effort to improve the quality of the experience of young drivers during the mandatory supervised driving period, a new program entitled “Green Light for Life” was conducted in 2005 in Israel. This program included a meeting with the young driver, his parents, and a supervisor, during which guidance was given regarding the best practices for the accompanied driving period, as well as advises for dealing with in-vehicle parent–teen dynamics. In order to evaluate the effectiveness of the program, Toledo et al. (68) compared official crash records of young drivers who participated in the program with crash records of all other license holders at the same period. The analysis indicated a significant 10% decrease of crash records for those who participated in the program, within 24 months after obtaining their driving license. Therefore, this program seemed to raise awareness to the importance of the accompanied driving phase in order to reduce the incidence of young drivers’ road accidents.

In addition, the GDL is unique in the history of road safety *via* its great impact on the targeted group, in one U.S. state, showing a steady decrease of teens drivers’ accident risks by at least 25% (69). The implementation of GDL programs could therefore save a substantial number of lives. In fact, several studies evaluating many angles of the GDL confirmed its effectiveness. In their study, Jones et al. (64) found that this program could prevent up to 114 deaths and 7,366 victims per year in Great Britain. According to Foss (70), improving the functioning of GDL programs would probably require a better understanding of adolescent driving behaviors.

Finally, according to Chen et al. (67), pediatricians and family physicians could play an important role in working with legislators to implement GDL programs by encouraging parents of young drivers to enforce the requirements of GDL.

#### Advertising Campaigns for Road Safety

Regarding road-related risks, many studies showed that the effectiveness of educational and preventive road safety programs is yet to be confirmed (71). Despite the popularity of road safety advertisements, speeding is still considered socially acceptable. The lack of efficiency in advertising campaigns could be due to the fact they are often based on intuition rather than principles of psychology. Thus, they seemingly fail to target the right factors (72). In fact, advertisement and educational campaigns in favor of road safety are primarily based on persuading drivers in complying with speed limits (by providing drivers, for example, with information about speeding consequences).

Although there is still no consensus on the effectiveness of road safety preventive strategies, research showed that advertising based on threats (highlighting dangers such as being injured or killed in a crash) was very effective (73). In fact, the technique incorporating physical threats of death and injuries in advertisements increased the intensity of emotional and cognitive responses compared to advertisements based on less threatening and more informative or humorous messages.

A study in Quebec conducted by Daignault and Paquette (74) also examined the effectiveness of threatening advertisements by evaluating television messages varying on 3° of realism (symbolic/realism/hyperrealism). They found that messages illustrating a threat in a hyperrealistic manner were the most effective at highlighting the importance of emotional and cognitive processes related to advertising information processing.

### Current Therapeutic Strategies

Twisk et al. (75) tested five road safety educational programs for pedestrians and cyclists. Three programs were based on a cognitive approach (road safety education, practical exercise, stimulation of empathy…), whereas the two others were based on fear or negative emotions. Young people, aged 12 and 18 years, were divided into five groups according to their age and each age group received one of the five programs. Results showed that the three programs based on road safety education significantly improved self-reported safety behavior. It would, however, be interesting to compare the effect of these educational programs on other groups of the same age.

Young people generally achieved a psychological balance in their lives by experimenting risk taking behaviors. This process is a personal exploration of their identity while seeking for their autonomy (14). Carbone (76) insisted on implementing psychological counseling for young drivers following an accident. This is mainly the reason why she proposed the inclusion of a support group for hospitalized adolescents after an accident. The author indicated that teens communicated their experiences of fear, loneliness, and danger. In fact, the experience of speaking in a peer group in the presence of a therapist confirmed the important need of these teenagers to express themselves while being supported by an adult.

### Preventing Recurrence of Traffic Accidents in At-Risk Adolescents and Young Adults: Protocol ECARR2

To compensate for the lack of effectiveness of road safety campaigns and considering the well-established interest in psychological group care during adolescence, we decided to evaluate an innovative strategy: a postaccident group therapeutic intervention aimed at preventing recurrence of traffic accidents. For this to happen, we proposed a randomized, multicenter, interventional, case–control study in the west of France.

Every teenager included in the experimental group would attend three group sessions 1 month after being involved in an accident. Each group session would be 1 week apart and would consist of integrative sessions combining motivational interviewing and cognitive-behavior therapy.

The ongoing study started in January 2016 and included 12 adults and pediatrics emergency departments in the West of France. Its main purpose is to show a minimum decrease, at 12 months after study inclusion, of 20% in the number of traffic accidents in a group of adolescents and young adults involved in a road accident and with a high risk of recurrence. To do so, we will compare two groups of patients at high risk of recurrence: a group receiving therapeutic prevention intervention and a control group. The intervention will consist of three sessions per week for three consecutive weeks. The inclusion period is estimated to last 1 year in order to include 300 participants, divided equally in the two groups mentioned above. Each patient will be followed up for 1 year after his/her initial inclusion in the study. In addition, all participants will be evaluated by phone at three (T3), six (T4), and 12 (T5) months after their inclusion.

Ultimately, from a public health perspective, proving the effectiveness of this program could allow it to be generalized in France.

## Conclusion

This work analyzes some factors involved in traffic accidents in youth. Two categories of factors seem to be associated with traffic accidents: (1) factors specific to the traffic environment and (2) “human” factors, which seem to be the most influential.

Some interventional driving strategies and preventive measures have reduced the risk of traffic accidents among young people; so far, few therapeutic approaches have been implemented. We decided to set up an innovative strategy consisting of a therapeutic postaccident group intervention, ECARR2 to prevent recurrence among adolescents and young adults identified at risk, taking into account the multiple risk factors.

## Author Contributions

LG determined the topic, managed this review, corrected, and translated it and is organizing the associated research. PO, EB, and CT contributed to bibliographic researches and to the redaction of the article. LR contributed to bibliographic research and redaction of the article.

## Conflict of Interest Statement

The authors declare that the research was conducted in the absence of any commercial or financial relationships that could be construed as a potential conflict of interest.
